# Pattern and Distribution of Colorectal Cancer in Tanzania: A Retrospective Chart Audit at Two National Hospitals

**DOI:** 10.1155/2016/3769829

**Published:** 2016-11-14

**Authors:** Leonard K. Katalambula, Julius Edward Ntwenya, Twalib Ngoma, Joram Buza, Emmanuel Mpolya, Abdallah H. Mtumwa, Pammla Petrucka

**Affiliations:** ^1^School of Life Science and Bioengineering, Nelson Mandela African Institution of Science and Technology, Arusha, Tanzania; ^2^Department of Public Health, University of Dodoma, Dodoma, Tanzania; ^3^Muhimbili University of Health and Allied Sciences, Dar es Salaam, Tanzania; ^4^Department of Statistics, University of Dodoma, Dodoma, Tanzania; ^5^Adjunct Faculty, Nelson Mandela African Institution of Science and Technology, Arusha, Tanzania; ^6^University of Saskatchewan, Regina, SK, Canada

## Abstract

*Background*. Colorectal cancer (CRC) is a growing public health concern with increasing rates in countries with previously known low incidence. This study determined pattern and distribution of CRC in Tanzania and identified hot spots in case distribution.* Methods*. A retrospective chart audit reviewed hospital registers and patient files from two national institutions. Descriptive statistics, Chi square (*χ* ^2^) tests, and regression analyses were employed and augmented by data visualization to display risk variable differences.* Results*. CRC cases increased sixfold in the last decade in Tanzania. There was a 1.5% decrease in incidences levels of rectal cancer and 2% increase for colon cancer every year from 2005 to 2015. Nearly half of patients listed Dar es Salaam as their primary residence. CRC was equally distributed between males (50.06%) and females (49.94%), although gender likelihood of diagnosis type (i.e., rectal or colon) was significantly different (*P* = 0.027). More than 60% of patients were between 40 and 69 years.* Conclusions*. Age (*P* = 0.0183) and time (*P* = 0.004) but not gender (*P* = 0.0864) were significantly associated with rectal cancer in a retrospective study in Tanzania. Gender (*P* = 0.0405), age (*P* = 0.0015), and time (*P* = 0.0075) were all significantly associated with colon cancer in this study. This retrospective study found that colon cancer is more prevalent among males at a relatively younger age than rectal cancer. Further, our study showed that although more patients were diagnosed with rectal cancer, the trend has shown that colon cancer is increasing at a faster rate.

## 1. Introduction

The rate of colorectal cancer (CRC) is increasing globally, although its ranking across all types of cancer has remained the same between 2008 and 2012, and its prevalence rose from 663,000 cases to 746,000 cases among males and from 570,000 cases to 614,000 cases among females [1, 2]. Currently, Australia/New Zealand and Western Europe were ranked on top of the list, while West Africa was at the bottom [3]. However, it is concerning that the upward trend is primarily observed in countries which are in economic transition.

According to data from Globocan, global cancer trends are estimated using incidence and 5-year relative survival or based on samples or based on neighboring countries' incidence and survival rates [3]. In Africa, CRC is the fourth most common fatal malignancy, although slight regional variation in prevalence has been reported. CRC ranks third amongst all types of cancers in North Africa, while in East, Central, South, and West Africa it is ranked fourth [3]. It is noted that many of these nations lack official cancer registries, which may reduce accuracy and reliability of the data. For example, the Tanzanian National Cancer Registry is at its infancy stage after failure of several attempts since 1963 [4]. At this point, in Tanzania, the prevalence of CRC was 1239 cases with a projected increase by 37.5% by 2035 [1].

### 1.1. Lifestyle Risk Factors for Colorectal Cancer

CRC incidence has risen dramatically in the last fifty years, especially in developing contexts [5] often related to risk factors such as adoption of westernized diets, obesity, and reduced physical activity [6, 7]. CRC has been called a disease of the environment as it is frequently evidenced in the second and third generations of migrants from low- to high-risk CRC incidence countries. This attribution to the environment is also evident through comparison of rural and urban dwellers and racially diverse people within the same nation, such as in South Africa, where there is a confirmed limited genetic contribution in the etiology of this disease [8, 9].

#### 1.1.1. Diet

Dietary factors account for between 30% and 50% of all CRC incidences [10]. Diet variably acts as a pro- and antitumor risk modifier across the CRC tumorigenesis process, which includes tumor initiation, promotion, and progression [11]. Developing countries undergo profound changes in societal and behavioral patterns, including shifting dietary patterns due to socioeconomic and cultural changes, globalization of food markets, urbanization, and economic growth [12]. In Tanzania, the nutrition transitions in recent years have contributed to low fiber, high sugar, and saturated fat-ladened diets often related to processed and packaged foods [13]. The exponential growth in availability of inexpensive calorie-ladened foods (i.e., cakes, sugary drinks, and chocolate) has led to gradients in energy intake between rural and urban populations [14]. Daily consumption by urbanites in Dar es Salaam was 1600 kcal for the low-income groups and 3000 kcal or more for the high-income group with most originating from fats (43%) [14]. Keding et al. [12] described five rural dietary patterns, with the “Purchase” pattern characterized by bread and cakes (usually fried), sugar, and black tea being the most prevalent pattern. The remaining four patterns included “Traditional-coast” (i.e., fruits, nuts, starchy plants, and fish), “Traditional-inland” (i.e., cereals, oils and fats, and vegetables), “Pulses” (i.e., pulses, with few or no vegetables), and “Animal Products” (i.e., meat, eggs, and/or milk) [12].

Epidemiological studies report a significant relationship between red meat consumption and CRC development [15, 16], yielding recommendation that it should be eaten less frequently or avoided. The mechanism suggested for red meat carcinogenesis includes exposure to different carcinogens produced by meat during the cooking processing and level of doneness [17]. Meat prepared at high temperatures and well done produces more heterocyclic amines compared to that prepared at lower temperatures [18]. Consumption of 50 grams of processed meat on a daily basis has been shown to increase the risk of CRC by 18% over the lifetime [19].

World Health Organization [WHO] [20] estimated that 14% of gastrointestinal cancer deaths are caused by insufficient intake of fruits and vegetables worldwide. A number of authors [19, 21–23] indicated that higher intake of vegetables probably lowers the risk of CRC. Reports from East African countries suggest lower consumption of fruits and vegetables [24] than the recommended daily 400 g [20]. Most people in Tanzania consume 164 g per day only, more than 200 g below the recommended dietary guidelines [24].

There is an evidentiary association between body mass index (BMI) and colon cancer [25]. Specifically, studies have shown that overweight and obesity are related to cancer of the colon and several types of cancer [22, 26, 27]. Obesity is associated with a 30–70% increased risk of colon cancer in men, but such association is not consistently reported in women [28]. Visceral fat, or abdominal obesity, is more predictive than subcutaneous fat obesity [29].

In a multicountry study of urban areas in Africa, analysis of BMI data on women found that the prevalence of overweight and obesity was higher than the prevalence of underweight in 17 of 19 countries [30]. In Tanzania, several studies assessing obesity have been done [30–34].

#### 1.1.2. Physical Activity

Studies with diverse populations showed that physically active individuals, especially lifelong adherents, are at a lower risk for CRC, an effect which was independent of other risk factors (i.e., diet and body weight) [35, 36]. Different types of physical activity, even a relatively moderate level of activity (i.e., walking fast for one hour daily), can reduce risk of colon cancer [37]. The level of physical inactivity varies between genders and between rural and urban residents in Tanzania with most reporting that urban dwellers and women were less active [34, 38] than their rural and male cohorts.

#### 1.1.3. Cigarette Smoking

Cigarette smoke contains over known 60 carcinogens and free radicals, which could affect the colorectal mucosa, thereby potentiating the alteration of cancer related genes [39]. The association between cigarette smoking and CRC depends on the number of cigarettes smoked, length of exposure, and age of initiation, which cumulatively yields a risk trajectory over an extended, continuous period [40]. Limited information is available regarding tobacco smoking in Tanzania, with an estimation of 18% of males and 1.3% of females as daily smokers in the age range of 25–64 [40].

#### 1.1.4. Alcohol

An association between alcohol consumption and CRC was reported in more than 50 prospective and case control studies, with no difference in the risk for colon and rectal cancers [41]. In a meta-analysis of eight studies of colon cancer, a combined relative risk (RR) of 1.09 (1.03–1.14) per 10 g intake per day [42] was reported, which was mirrored in the meta-analysis findings (RR 1.06 (1.01–1.12) per 10 g daily intake) of the meta-analysis by McMichael [43].

#### 1.1.5. Risk Factors for Colon and Rectal Cancer Subsites

Studies have suggested different etiologies for colon and rectal cancer subsites [44–46]. Although the findings are not conclusive, evidence has linked colon cancer with lifestyle factors (i.e., physical inactivity, body mass index (BMI), and gender) [47–49] more than rectal cancer. For example, physical inactivity and body mass index (BMI) have been associated strongly with colon cancer but not with rectal cancer [50]. Conversely, several studies found no association between body weight and BMI with rectal cancer [51]. Still other studies have indicated no or slight difference in risk between the two types of cancer [5, 49].

### 1.2. Age and Gender in Relation to CRC

Although CRC can occur at any age, the chances of developing the disease increase with age and peak after the age of fifty [52]. Of note, age-gender difference in CRC incidence exists with women developing CRC at an older age than men [53, 54]. In general, a four-to ten-year age difference by gender has been reported, with female incidence higher in the age range of 70–74 and male incidence higher in the age range of 60–64 [55, 56]. In most developing countries, diagnosis occurs at a relatively younger age than in developed countries [57].

Age distributions must be a consideration in the predictions of current and future prevalence of CRC.

### 1.3. Accessibility of Health Services for Diagnosis

For CRC, health care accessibility is important for early detection and treatment. People in low-to-middle income countries have less access to health services than those in developed countries [58]. Several barriers to health service accessibility are reported in the literature, which have been categorized at the patient level, provider level, and system level [59]. At the patient level, barriers relate to individual traits (i.e., sex, ethnicity, and income); at the provider level to services characteristics (i.e., skills and attitudes); and at the system level traits to broad factors (i.e., policy and organizational factors).

### 1.4. Summary

Adoption of cancer risky lifestyles has been associated with a rapid increase in cancer incidence in Africa [60]. In general, dietary patterns (i.e., high consumption of red meat and high caloric foods) and lifestyle choices (i.e., alcohol, smoking, and sedentary lifestyle) are significantly associated with an increased risk of CRC [7, 10, 13, 25, 37, 51]. Demographic risk factors have also been linked to CRC including being male, increased age, and low socioeconomic status [61]. High consumption of fiber, fruits, and vegetables is associated with protective roles against colorectal cancer [62, 63]. Lack of access to health care services may also influence pattern and distribution of colorectal cancer [58, 59].

In light of these complex and emergent issues, the aim of this study was to determine the patterns and distribution of known colorectal cancer cases in Tanzania in order to inform future policy planning and the design of targeted interventions.

## 2. Materials and Methods

### 2.1. Study Area

Tanzania is situated in East Africa sharing borders with Kenya and Uganda to the North, Rwanda, Burundi, and the Democratic Republic of Congo to the West, and Zambia, Malawi, and Mozambique to the South with the Indian Ocean forming its eastern border [64]. As of the 2012 census, Tanzania's population was nearing 45 million with 43,625,354 on Tanzania mainland and 1,303,569 on Zanzibar islands [64]. Thirty administrative regions make up the country; 25 regions are found on the mainland and 5 in Zanzibar [64]. Zanzibar was grouped as one region for the purpose of this study. Tanzania's population is comprised primarily of native Africans (99%) utilizing more than 120 local languages. The majority of people follow Christian and Muslim religions, although there are small numbers following Hinduism and other faiths [64]. The economy is reliant upon agriculture, contributing to more than 27% of the Gross Domestic Product and accounting for 80% of the total workforce [64].

### 2.2. Study Sites

This study was conducted between September and November 2015 in two national hospitals [Muhimbili National Hospital (MNH) and Ocean Road Cancer Institute (ORCI)], both based in Dar es Salaam. Established over a century ago, MNH is a university teaching hospital and the only national referral hospital. Currently, MNH has a bed capacity of 1,500, serving 1,000 to 1,200 outpatients per week, as well as admitting approximately the same number weekly [65]. The major role of MNH in cancer care and treatment is to receive patients from all parts of the country who need further investigation and treatment plus providing referral as appropriate for radiation treatments to ORCI. ORCI is the only specialized facility for cancer treatment in Tanzania. The facility was established in 1895 by the German colonial government. In 1996, it was made an independent autonomous institute by an Act of Parliament. ORCI offers patient services including laboratory services, diagnostic imaging, chemotherapy, radiotherapy, palliative care services, cervical cancer screening, and an HIV/AIDS care and treatment clinic [66]. These hospitals (MNH and ORCI) are the two major hospitals for diagnosis and treatment of cancer in Tanzania.

### 2.3. Study Design

This study employed a retrospective chart audit method approach which collects codes and analyzes data that were originally collected for nonresearch purposes, such as admission and discharge documentation, as well as laboratory and diagnostic testing reports [67].

#### 2.3.1. Data Collection

Data collection involved all available patient charts for the period between January 2005 and November 2015. Review of patient files was done at MNH. File numbers were generated via the hospital's computer system with records from January 2005 to November 2015 identified with the actual files retrieved by medical record staff. A total of 704 records were found in the computer system; however, the file search retrieved 366 files. Data cleaning removed 59 files that were incorrectly recorded, presented unconfirmed diagnosis, and/or had important information missing. The status of most missing files was attributed to the fact that patients referred to ORCI physically carry their MNH files, which then remain at ORCI unless the patient is rereferred to MNH.

At ORCI, the new patients register book and, when necessary, patient files were used to collect information. All register books from 2005 to 2015 were found except that of 2007 which was reported as unaccounted for. Records of patients from MNH and from other parts of the country were located. For patients coming from MNH, we recorded the original MNH file number which was then used to remove duplicates from those found at MNH by using the Excel™ computer software program. Finally, 901 charts were included in this study (see Table 1 for the breakdown).

#### 2.3.2. Inclusion and Exclusion Criteria

This study included all patient charts indicating a diagnosis of CRC in accordance with the International Statistical Classification of Diseases and Related Health Problems 10th Revision (ICD-10) WHO Version for 2016. This categorization considers the relevant elements of the C18 (malignant neoplasm of colon) palette, such as C18.0, C18.2, and C18.4; C18.6, C18.7, C18.8, C18.9, and C19; and C20, C21, and C21.1. Any incomplete (missing diagnosis, i.e., gender, age, gender, and residence) or wrongly categorized charts were not retrieved for the purpose of this analysis.

#### 2.3.3. Data Collection Tool and Techniques

A customized retrospective audit form was used to collect information from patient records. File number, age, gender, and geographical region where the patient is coming from were all recorded. To avoid recording patients' temporary residences, we used referral letters to locate original residences. Also, we were aware that the referral hospital may not reflect the area of residence as some patients seek medical attention from hospitals which they trust; so the study used any other information such as patient history to determine the region of primary residence.

Data extraction was done by the principal investigator and a trained nurse. Data were extracted digitally with single extraction only. To ensure data integrity, the research assistant was trained and the principal investigator remained available for consultation throughout the data extraction period. Further screening was done during data analysis with all charts which did not meet the inclusion criteria being removed from the analysis.

#### 2.3.4. Standardization

Age standardization of incidence rates was done by using the direct method and age specific rates for 5-year age groups using the world standard population [68].

### 2.4. Data Analysis

The data abstracted from the records were entered in an Excel file, edited for consistency, and analyzed by Statistical Analysis System (SAS) Software™ (Version 9.4) as well as R software. During analysis of CRC subsites, patients with rectosigmoid cancer were excluded because our interest was on colon and rectal cancers only. Four patients of age below 15 years were excluded because the number was too small and could have affected the analysis. Thus, a total of 822 subjects were included in the analysis. Descriptive statistics were used to determine patterns and distribution according to age, gender, tumor site, and region of primary residence. Heat and contour maps showing distribution of cases were drawn to identify the most affected regions. Chi square (*χ* ^2^) tests were used to determine association between categorical independent variables and the response variable. To estimate the gender difference in prevalence of CRC, crude relative risk (RR) with a 95% confidence interval was calculated using the univariate log-binomial regression model. Multivariable log-binomial regression analysis was employed to determine how gender is associated with risk of having either colon or rectal cancer; the association was adjusted for age of the patients and the time difference (years) from the starting period of the study. *P* values were estimated by two-sided tests. Statistical significance was set at *P* value of less than 0.05.

### 2.5. Ethical Considerations

This study received ethical approval from the MNH Institutional Review Board (permission number: 625) and approval by the National Institute of Medical Research Ethical Committee. No client consent was required as this was a historical, retrospective chart review.

## 3. Results

### 3.1. Distribution of Colorectal Cancer Incidence by Hospital and Gender

**Figure 1:**
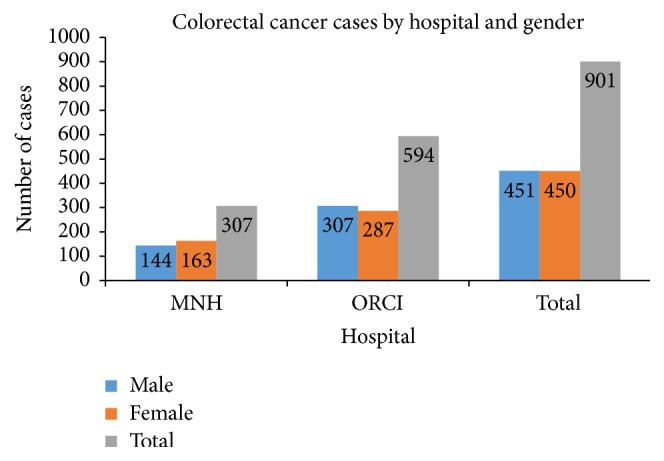
CRC cases distribution by gender and hospital.

CRC cases are almost equally distributed between the two genders (see Figure 1). Within the 901 charts reviewed, 451 (50.1%) were male patients and 450 (49.9%) were female patients. More than 65% of patient's charts were retrieved from ORCI. At ORCI, male patients (307) represented 51.6% of the total. About one-third of patients charts included in this study were recorded at MNH. The majority (53%) of patients at MNH were female (163).

### 3.2. Distribution of Colorectal Cancer Incidence by Age in Relation to Gender

**Figure 2:**
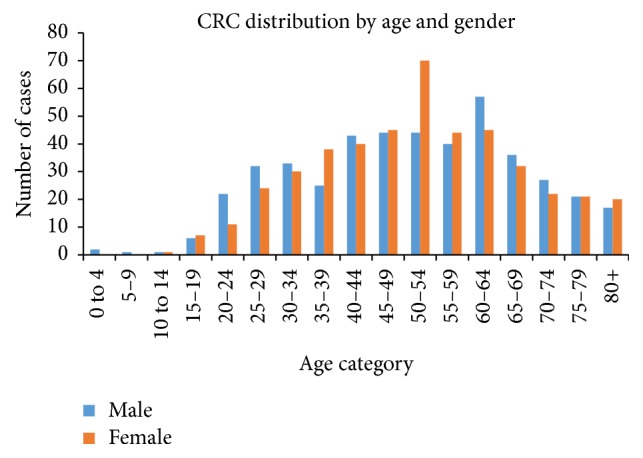
CRC cases distribution by age and gender.

Figure 2 shows the age and gender distribution of colorectal cancer patients whose charts were reviewed. The range in years was from 3 to 108 with the majority of males being at the age of 60–64 (12.6%) and the majority of females being at the age of 50–54 (15.6%). People aged 0–39 years comprised almost one-quarter of all patients (26%). Patients aged 40 to 69 represented the largest proportion at 62%. However, the Cochran-Mantel-Haenszel statistical test for association between age and sex of CRC patients was not statistically significant (*P* = 0.724).

### 3.3. Distribution of Colorectal Cancer Incidence by Geographical Area

**Figure 3:**
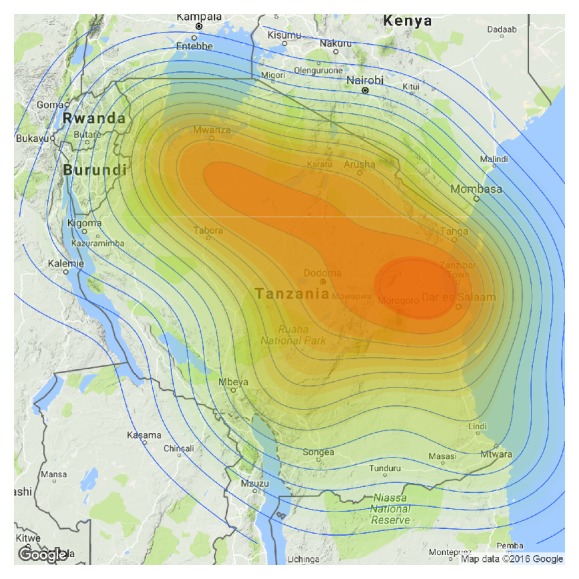
Geographic distribution of colorectal cancer.

**Figure 4:**
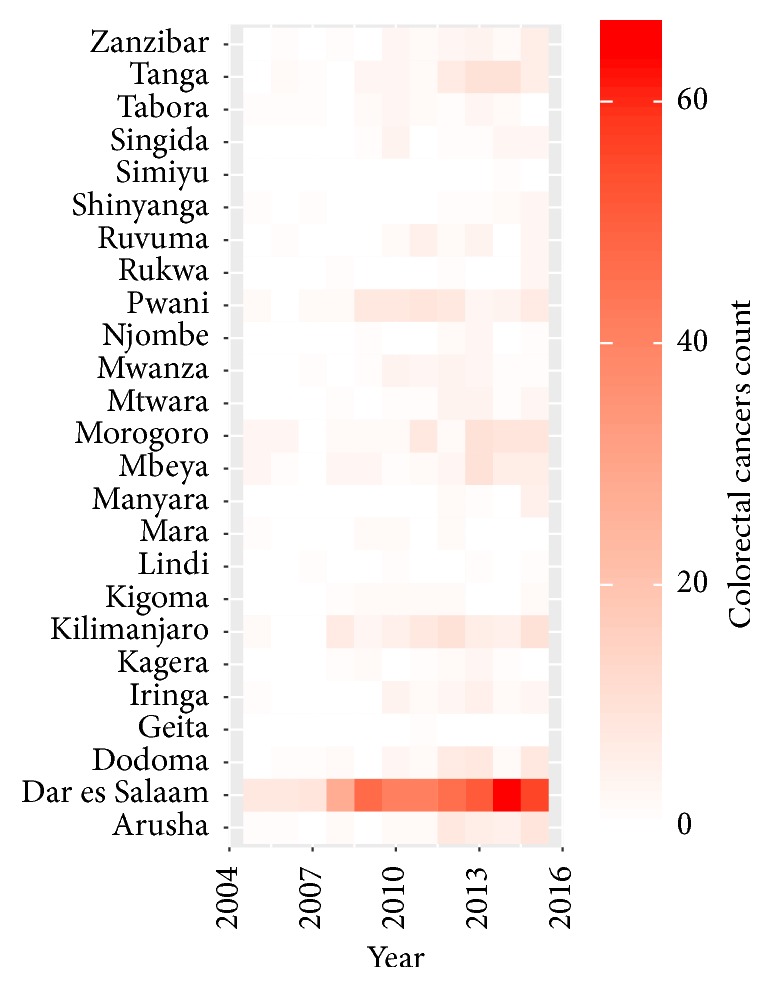
Colorectal cancer cases by region and year.

The number of CRC patients by region is visualized in Figures 3 and 4. Eastern zone showed high occurrence of the disease followed by the Northern, Central, and North West zones. Southern and Southern Highlands's zones showed lower occurrence (Figure 3). The number of patients (shown as density) is shown by shift in color from green (low intensity) to red (high intensity) colors as shown on the scale.

### 3.4. Distribution of Colorectal Cancer Incidence by Region and Year of Diagnosis

About 406 (45.1%) of all patients reported Dar es Salaam region as their primary residence. Since 2005, the number of CRC cases in Dar es Salaam was higher compared to other regions. The peak number was recorded in 2014, although, since 2008, there was an annual observable increase (Figure 4). The distribution of CRC cases by region and year of diagnosis is shown in Figure 4. *y*-axis shows regions of residence of patients and *x*-axis shows the year of diagnosis. The number of patients is plotted in white-to-hot red color scale. The hot red color of the tiles indicates high abundance and white color indicates low abundance.

After standardization of age to obtain the age adjusted incidence rates (ASR) (standardized to the world standard population), the ASR varied more than 100 times among the 25 populations analyzed. The highest rate was recorded in Dar es Salaam (20.2 per 100,000) and the lowest rate was recorded in Geita (0.1 per 100,000).  Table 2 reports the ASR for all regions.

### 3.5. Trends of Colorectal Cancer by Year

**Figure 5:**
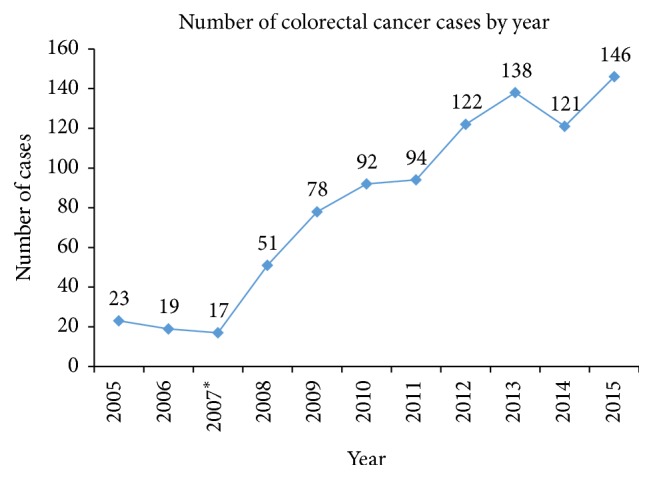
CRC cases distribution by year. *∗* shows that there were missing records in that year from Ocean Road.

There is a positive growth trend of CRC patients from 2005 to 2015. Despite missing data in 2007 and nongrowth in 2006 and 2014, the number of CRC patients increased continually from 23 patients in 2005 to 146 patients in 2015. There has been a sixfold increase in CRC incidence in a period of eleven years (see Figure 5).

### 3.6. Distribution of Colorectal Cancer Incidence according to CRC Subsite Affected

**Figure 6:**
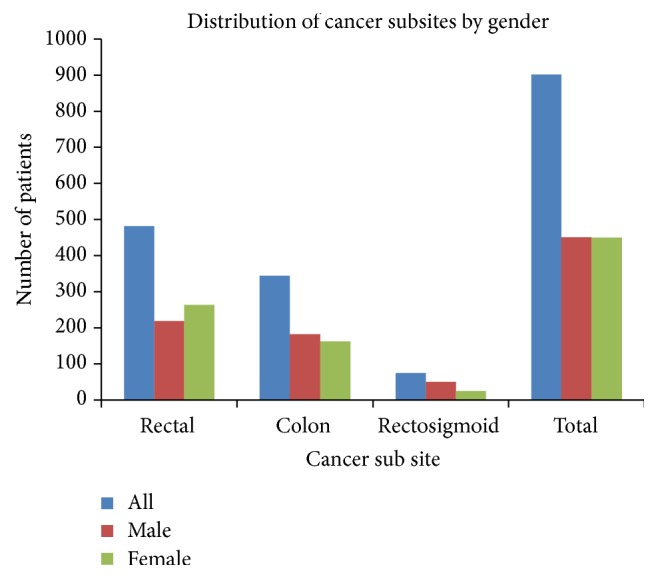
CRC cases distribution by subsite and gender.

Among the 901 charts included for analysis, cases of rectal cancer (482) were more common compared to colon cancer (344) and rectosigmoid junction cancer (75). Females were diagnosed more often with rectal cancer than their male counterparts (265 : 219), while males were diagnosed more often with colon cancer (162 : 182). More than two-thirds of patients (50) with rectosigmoid junction cancer were males (see Figure 6).

### 3.7. Distribution of Two Major Subsites (Colon and Rectal) Cancer by Gender and Age

Out of the 822 subjects to be considered, 478 (58.15%) and 344 (41.85%) were reported to have rectal and colon cancer, respectively.  Table 3 summarized the percentage distribution of CRC subsites by gender and age of patients. There was a significant difference between males and females in the probability of getting either colon or rectal cancer (*P* = 0.027). With regard to age, rectal cancer was found to be common in older adults such that patients aged 80+ years were more susceptible to rectal cancer (78.13%) followed by patients of age of 50–54 (65.71%). On the other hand, patients of young age category of 20–24 (54.84%) and 15–19 (53.85%) were more susceptible to colon cancer as compared to subjects in other age categories but the differences were not statistically significant (*P* = 0.3744) as shown in Table 3.

### 3.8. Gender, Age, and Time in Relation to Colon Cancer Incidence

The results of univariate analysis for colon cancer (Table 4) show that there is significant gender difference (*P* = 0.0428) in risk of having colon cancer, with males being at greater risk. Adjusting for age and time (years), the results of the fitted multivariable log-binomial regression model confirmed that male patients were 1.2 times more likely to develop colon cancer than their female counterparts [ARR = 1.203; 95% CI: 1.02, 1.41]. The risk of having colon cancer was significantly higher in people below 35 years. People aged 15–19 (*P* = 0.0325), 20–24 (*P* = 0.0134), 25–29 (*P* = 0.0219), and 30–34 (*P* = 0.042) had higher risk of developing colon cancer as compared to older patients (80+ years). Higher risk was also observed in people aged 65–69 (*P* = 0.035). People aged 20–24 (ARR = 2.43; 95% CI: 1.18, 5.01) were 2.4 times more likely to be diagnosed with colon cancer as compared to older patients (80+ years).

### 3.9. Gender, Age, and Time in Relation to Rectal Cancer Incidence

With respect to time difference, increase in time (in years) was significantly associated with an increased likelihood of having colon cancer (*P* = 0.0052). Results of multivariable analysis revealed that there is significantly positive association between time and risk of having colon cancer (ARR = 1.05; 95% CI: 1.01, 1.08); with one-year increase, the chance of diagnosing a colon cancer case increased by 2 percent (Table 4).

The results of univariate analysis (Table 5) indicated that gender, time, and age were significantly associated with risk of rectal cancer, with males having lower chance of developing rectal cancer (RR = 0.88; 95% CI: 0.78–0.99) than females. In the multivariable analysis, after controlling for age and time, gender was no longer significantly associated with risk of rectal cancer (*P* = 0.098). With exception of patients in age of 15–19 (ARR = 0.1238; 95% CI: 0.34–1.14), 40–44 (ARR = 0.79; 95% CI; 0.61, 1.01), and 50–54 (ARR = 0.84; 95% CI; 0.67, 1.040), patients in other age categories were significantly less likely to have rectal cancer as compared to patients with 80+ years of age. Increase in time (years) was significantly associated with decreased likelihood of diagnosing a new rectal cancer patient (ARR = 0.97; 95% CI: 0.95–0.99). With increasing time the chance of diagnosing rectal cancer decreased by 1.5% per year.

## 4. Discussion

### 4.1. Pattern and Distribution of CRC Incidence in Tanzania

In previous years, nutritionists in developing countries have focused on childhood malnutrition, protein energy malnutrition, and how to feed the world's population [69]. Currently, WHO estimates that 38 million people die annually from noncommunicable diseases (NCDs) including cancer with almost three-quarters of these deaths occurring in low-income and middle-income countries [70].

In this study, we have seen that CRC incidence rates are generally increasing for both males and females. A sixfold increase has been seen in a period of eleven years. One-quarter of CRC patients were below 40 years of age, with no discernable difference in the distribution of CRC cases among males and females. The majority of females are diagnosed at earlier age than men. Regional distribution shows that Dar es Salaam was disproportionately over represented for CRC in comparison to other regions.

Multiple factors acting either singly or in combination may be responsible for the trends in incidence and distribution of CRC patients in Tanzania. Epidemiological studies are highly suggestive of a direct correlation between the incidence of CRC and several lifestyle factors [27, 37, 69].

Physical inactivity and excess body weight are two modifiable and interrelated risk factors. It is recommended to be physically active and to avoid overweight and obesity in order to prevent CRC [49]. Higher levels of physical activity are associated with a lower risk of CRC, although the evidence is stronger for colon cancer than for rectal cancer [70]. The proposed biologic mechanisms of physical activities in reducing CRC risk include raised metabolic rate and increased maximal oxygen uptake [71]. Long-term and regular physical activities increase the body's metabolic efficiency and capacity, as well as reducing blood pressure and insulin resistance [72]. In Tanzania, the level of physical inactivity has been reported to vary between genders and between rural and urban residents. Studies have reported low physical activity levels in urban areas compared to rural areas [38] as well as lower levels low in women compared to their male counterparts [39].

The lack of physical activity has been attributed to the increased incidence of obesity in men and women, another factor associated with CRC [69]. Obesity has been linked to CRC in several studies [25–27, 69]. The greater future burden of obesity and noncommunicable diseases was predicted to affect developing countries including the world's poorest countries, especially urban areas [73]. Several biologic mechanisms have been suggested to explain the association between obesity and CRC. Circulating estrogens and decreased insulin sensitivity as a result of abdominal adiposity were related to increased CRC levels [72]. Together with excess energy intake, metabolic inefficiency has been cited to cause increase in overweight and obesity [74]. Prevalence of obesity is high in Tanzania, especially among women and urbanites and people with high socioeconomic status [34, 35]. Dar es Salaam records the highest prevalence of obese people [33, 34] compared to other cities [12, 30]. The rates, especially among women, are almost comparable to those reported in the United States [75].

Another factor for consideration is alcohol consumption. Regular consumption of alcohol is associated with increased risk of developing CRC [76]. Younger age onsets as well as increase of tumors in the distal colon are also associated with alcohol consumption [76–78]. Reactive metabolites of alcohol such as acetaldehyde are suspected to be carcinogenic [77]. Additionally, alcohol has been linked to the production of prostaglandins, lipid peroxidation, and the generation of free radical oxygen species [77]. Tanzania reports the prevalence of current alcohol consumers to be between 23% and 37% in males and between 13% and 23% in females [79]. More than 40% of adults in Tanzania consume alcohol [45] with local brews accounting for about 86% of all alcohol consumed [79].

Some authors have suggested the possibility of change in dietary pattern and nutrition transition as the factor for increase in CRC incidence [80, 81]. Diets high in fat, especially animal fat, and low in fibers are a major risk factor for CRC [81]. Fat is implicated in favoring the development of a bacterial flora capable of degrading bile salts to potentially carcinogenic compounds [82]. High meat consumption has also been associated with development of CRC [19]. Meat consumption is strongly positively associated with colon cancer compared to rectal cancer [82]. Possible mechanisms for a positive association of red meat consumption with CRC include the presence of heme iron in red meat and production of heterocyclic amines and polycyclic aromatic hydrocarbons believed to have carcinogenic properties as a result of cooking meats at high temperatures [83, 84].

In addition, WHO and other international organizations and researchers have suggested that people who eat less fruits and vegetables are at higher risk of CRC [20, 21, 23].

Smoking is harmful to the colon and rectum [77] with the carcinogens found in tobacco linked to increasing risk of being diagnosed with CRC and cancerous growth in the colon and rectum [85]. It has been reported that cigarette smoking facilitates formation and growth of adenomatous polyps [85]. Young age onset of CRC is also linked to cigarette smoking [77, 78]. Studies show that 35% of adults smoke regularly in Tanzania and 32% of all cancers at one institute in Dar es Salaam were attributed to tobacco use [86]. Almost 20% of Tanzanian males and less than 2% of females aged 25–64 are current smokers [40].

Gender difference in CRC epidemiology has been established in several studies. Our findings resemble those reported in low incidence populations, such as Thailand, India, and Chile [87]. Conversely, our findings differ from the work of Chalya et al. [88] and other studies done in Africa where males were the primary CRC group [89–91]. These latter findings are similar to findings from those seen in high-risk populations and, of note, in previously low-risk populations currently experiencing a rising CRC incidence (i.e., Japan, Hong Kong, and Singapore) [80]. The global cancer estimates suggested that there are more females with CRC than males in developing countries [3], which may be attributable to methodological differences.

Regarding gender-age at diagnosis, our findings contradicted those previously reported [56], where females were reported to be diagnosed at older ages than their male counterparts [51]. The above discussed factors could partially explain why CRC incidence has been increasing in the previous eleven years in Tanzania. However, other considerations, such as patient awareness and improved data capture, should be given equal weight (i.e., electronic records at MNH). Other factors such as availability of and accessibility to health services improved diagnostic methods at MNH, ORCI, and other referral hospitals.

### 4.2. Distribution of Colorectal Cancer by Subsite

In this study, more cases of rectal cancer were reported compared to colon cancer, a finding similar to those in other African countries [86] but differing from a Tanzanian study [88] which showed prevalence of rectosigmoid cancer in comparison to other CRC subsites.

An interesting observation from this study was the decreasing trend in rectal cancer and increase in colon cancer. Rectal cancer was decreasing at an average of 1.5% per year [ARR = 0.97; 95% CI: 0.95, 0.99], while colon cancer was increasing at an average of 2% per year [ARR = 1.05; 95% CI: 1.01, 1.08]. Changes in environmental factors as discussed above may be shaping colorectal cancer distribution in Tanzania.

In this study, we found that gender had no influence on rectal cancer despite being more common among females in the last decade. However, gender is a significant determinant for developing colon cancer with males having more chance than females [ARR = 1.18; 95% CI: 1.01–1.39]. Our findings differ from those observed by Curado, et al., [90] where, in low incidence populations, sex ratio between these two subsites was comparable but rectal cancer was consistently higher among males than colon cancer in high incidence countries.

We also found that rectal cancer was more common to people of older age compared to young people, while colon cancer was more common to young adults. People aged below 40 years were more likely to develop colon cancer than people with 80+ years. This is contrary to what was variously reported by Rosato et al., [91] where rectal cancers occurred more in younger patients, while colon cancer occurred in patients on average a decade older. Lifestyle factors, especially alcohol consumption and cigarette smoking, are associated more with young onset of CRC [78, 87, 88].

## 5. Conclusions

CRC cases in Tanzania have shown an upward trend for the period from 2005 to 2015. Rectal cancers are most prevalent among the Tanzanian population compared to colon cancer. Colon cancer is increasing at higher rate than rectal cancer. Females are diagnosed at relatively younger age than males. The population category belonging to 50 to 54 years among females and 60 to 64 years among males was the peak age. CRC was equally distributed among males and females. Colon cancer occurs most among the young population, while rectal cancer was diagnosed more among older adults. Major towns and cities of Dar es Salaam, Pwani, Kilimanjaro, Arusha, Morogoro, Tanga, and Dodoma had the highest share of CRC patients. Gender of an individual significantly predicted the occurrence of colon and not rectal cancer. As we have seen, change in lifestyle can account in whole or in part for the observed trend shift between colon and rectal cancers in Tanzania. As a result, future research directions should include population level longitudinal studies.

## Figures and Tables

**Table 1:** Number and source of patient charts.

Source of record	Number of charts in the record	Retrieved charts	Removed charts	Total
Patient charts retrieved from MNH	704	366	59	307
Patients charts retrieved from ORCI with referral letter from MNH	286	286	0	286
Patients charts retrieved from ORCI with referral letter from other hospitals	308	308	0	308

**Table 2:** Age-standardized incidence rates per 100,000 (world standard) in Tanzania and different administrative regions of Tanzania.

Geographical area	Adjusted rates	Crude rates
Tanzania	3.831458994	64.92886737
*Arusha*	*4.192229604*	*73.32859002*
*Dar es salaam*	*20.23704632*	*330.2030566*
Dodoma	2.656542926	46.61069949
Geita	0.142077125	2.646692957
*Iringa*	*3.460510783*	*60.68991432*
Kagera	0.842052764	13.31652511
*Kilimanjaro*	*4.441958929*	*79.25571383*
Kigoma	0.945466469	13.31267019
Lindi	0.718486048	15.18403374
Mara	0.841284682	15.30577802
Manyara	1.050908994	15.53281304
Mbeya	2.485941105	41.43108835
*Morogoro*	*3.596505243*	*61.36434016*
Mtwara	1.660324899	30.25055109
Mwanza	1.590799707	28.81504262
Njombe	1.762859356	33.75304226
*Pwani*	*7.217865267*	*125.8673874*
Rukwa	1.227169289	19.57289293
Ruvuma	1.936379494	33.37093399
Shinyanga	1.088767012	20.89590056
Simiyu	0.156195397	4.200268817
Singida	1.845545557	31.07724838
Tabora	1.363903515	25.61944678
*Tanga*	*3.501658102*	*60.94362847*
Zanzibar	2.986722615	58.59521303

**Table 3:** Percentage distribution of colon and rectal cancer by gender and age.

Variable	Total frequency *n* (%) = 822	Rectal cancer *n* (%) = 478 (58.15)	Colon cancer *n* (%) = 344 (41.80)	*P* value
*Gender*				
Male	398 (48.36)	216 (54.27)	182 (45.73)	0.0270
Female	425 (51.64)	262 (61.88)	162 (38.12)	
*Age (years)*				
15–19	13 (1.58)	6 (46.15)	7 (53.85)	0.3744
20–24	31 (3.77)	14 (45.16)	17 (54.84)	
25–29	54 (6.57)	27 (50)	27 (50)	
30–34	59 (7.18)	32 (54.24)	27 (45.76)	
35–39	61 (7.42)	35 (57.38)	26 (42.62)	
40–44	78 (9.49)	47 (60.26)	31 (39.74)	
45–49	77 (9.37)	47 (61.04)	35 (38.96)	
50–54	105 (12.77)	69 (65.71)	36 (34.29)	
55–59	77 (9.37)	43 (55.84)	34 (44.16)	
60–64	94 (11.44)	56 (59.57)	38 (40.43)	
65–69	62 (7.54)	33 (53.23)	29 (46.77)	
70–74	42 (5.11)	23 (54.76)	19 (45.24)	
75–79	37 (4.50)	21 (56.76)	16 (43.24)	
80+	32 (3.89)	25 (78.13)	7 (21.88)	

**Table 4:** Crude and adjusted relative risk of colon cancer among CRC patients.

Variable	Univariate analysis			Multivariable analysis		
	RR	95% CI	*P* value	ARR	95% CI	*P* value
*Gender*						
Male	1.2027	[1.02, 1.41]	0.0252	0.18	[1.01, 1.39]	0.0428
Female	Reference					
*Age (years)*						
15–19	2.46	[1.08, 5.62]	**0.0325**	2.36	[1.04, 5.35]	0.0408
20–24	2.51	[1.21, 5.19]	**0.0134**	2.43	[1.18, 5.01]	0.0162
25–29	2.29	[1.13, 4.64]	**0.0219**	2.19	[1.09, 4.43]	0.0286
30–34	2.09	[1.03, 4.26]	**0.042**	2.07	[1.02, 4.20]	0.0441
35–39	1.95	[0.95, 3.99]	0.068	1.98	[0.97, 4.05]	0.0604
40–44	1.82	[0.89, 3.69]	0.0991	1.83	[0.91, 3.72]	0.092
45–49	1.78	[0.87, 3.63]	0.1121	1.75	[0.86, 3.56]	0.1199
50–54	1.57	[0.77, 3.18]	0.2124	1.60	[0.79, 3.24]	0.1894
55–59	2.02	[1.00, 4.07]	0.0496	2.03	[1.01, 4.08]	0.0471
60–64	1.85	[0.92, 3.72]	0.0852	1.87	[0.93, 3.76]	0.0772
65–69	2.14	[1.05, 4.33]	**0.035**	2.04	[1.01, 4.12]	0.0473
70–74	2.07	[0.99, 4.31]	0.0525	2.03	[0.98, 4.21]	0.0575
75–79	1.98	[0.93, 4.19]	0.0756	1.88	[0.89, 3.98]	0.0983
80+	Reference					
Time (years)	1.05	[1.01, 1.08]	0.0052	1.05 (0.02)	[1.01, 1.08]	0.0075

Note: ARR refers to adjusted relative risk and SE stands for standard error of relative risk.

**Table 5:** Crude and adjusted relative risk of rectal cancer among CRC patients.

Variable	Univariate analysis			Multivariable analysis		
	RR	95% CI	*P* value	ARR	95% CI	*P* value
*Sex*						
Male	0.88	[0.78, 0.98]	0.02480	0.91	[0.81, 1.02]	0.098
Female	Reference					
*Age (years)*						
15–19	0.59	[0.32, 1.09]	0.0935	0.62	[0.34, 1.14]	0.1238
20–24	0.58	[0.38, 0.89]	0.0123	0.59	[0.39, 0.91]	**0.0167**
25–29	0.64	[0.46, 0.88]	0.0069	0.65	[0.47, 0.90]	**0.0088**
30–34	0.69	[0.52, 0.93]	0.0162	0.70	[0.52, 0.94]	**0.0173**
35–39	0.73	[0.55, 0.98]	0.0329	0.74	[0.56, 0.97]	**0.0316**
40–44	0.77	[0.60, 1.00]	0.0477	0.79	[0.61, 1.01]	0.0608
45–49	0.78	[0.60, 1.01]	0.0587	0.78	[0.61, 1.00]	**0.0472**
50–54	0.84	[0.67, 1.06]	0.1397	0.83	[0.67, 1.04]	0.1041
55–59	0.71	[0.55, 0.94]	0.0149	0.73	[0.56, 0.95]	**0.0212**
60–64	0.76	[0.60, 0.98]	0.0319	0.79	[0.62, 1.00]	0.0515
65–69	0.68	[0.51, 0.92]	0.0113	0.69	[0.51, 0.92]	**0.0106**
70–74	0.70	[0.50, 0.98]	0.0351	0.71	[0.51, 0.98]	**0.0358**
75–79	0.73	[0.52, 1.02]	0.0621	0.71	[0.51, 0.99]	**0.0431**
80+	Reference					
Time (years)	0.97	[0.95, 0.99]	0.0069	0.97	[0.95, 0.99]	0.0047

## Abbreviations

ARR: Adjusted relative risk
CRC: Colorectal cancer
CI: Confidence interval
ASR: Age standardized rate
MNH: Muhimbili National Hospital
ORCI: Ocean Road Cancer Institute
RR: Crude relative risk
SE: Standard error
WHO: World Health Organization
WHO/FAO: World Health Organization/Food.

## Additional Points

*Limitations of the Study*. Retrospective data depend on clinician legible documentation; one major limitation was the lack of record intactness or completeness. Although quality assurance measures were in place including extractors training, using standardized forms and maintaining availability of the principal investigator for consultation throughout the data extraction period, medical record abstraction techniques can differ among extractors. Interrater reliability was not calculated, and this is a limitation.
